# BeetleAtlas 2: An enhanced *Tribolium castaneum* web resource for tissue and developmental transcriptomics allowing refinement of gene predictions

**DOI:** 10.1371/journal.pcbi.1014314

**Published:** 2026-06-01

**Authors:** David P. Leader, Muhammad T. Naseem, Janina L. Rinke, Kenneth Veland Halberg

**Affiliations:** 1 Institute of Molecular Cell and Systems Biology, College of Medical, Veterinary and Life Sciences, University of Glasgow, Glasgow, United Kingdom; 2 Department of Biology, Section for Cell and Neurobiology, University of Copenhagen, Copenhagen, Denmark; 3 Institute for Evolution and Biodiversity, University of Münster, Münster, Germany; University of Helsinki: Helsingin Yliopisto, FINLAND

## Abstract

*BeetleAtlas* is an online resource for tissue- and stage-specific transcriptomics in the red flour beetle, *Tribolium* *castaneum*. On updating from the original Tcas5.2 genome assembly to the more recent improved icTriCast1.1 genome assembly it became evident that there were major discrepancies between the gene models of the two genome annotations in use: the OGS3 and the NCBI gene sets. As neither was clearly superior we implemented a new design in *BeetleAtlas 2* (beetleatlas.org) comprising two parallel ‘modes’ — one incorporating results using the NCBI gene models and a second incorporating those using the OGS3 gene models. This allows direct comparison where equivalent gene models exist: 50–57% of cases. To aid resolution of discrepancies between the two gene model sets and verification of results, gene models are linked to a custom visualization of RNA-seq read coverage of the genome in the UCSC Genome Browser. This displays reads from 22 tissues and life stages superimposed on the icTriCast1.1 genome assembly. Reference tracks show the NCBI gene models, the OGS3 gene models after translation of their coordinates from the Tcas5.2 assembly, and 1050 discontinued NCBI gene models from the previous assembly after a similar transfer of coordinates. We document various situations in which distinct patterns of expression of the tissues can be used to confirm and extend correlations between the two gene sets, resolve discrepancies between them, make corrections and identify putative genes or exons absent from the current gene sets. *BeetleAtlas 2* allows those involved in *Tribolium* research to avoid the pitfalls inherent in incorrect gene models when planning experiments on specific genes and interpreting the results. It also demonstrates how *BeetleAtlas 2* might play an important role in establishing a revised gene set for *Tribolium castaneum* in the future.

## Author summary

We developed the web application, *BeetleAtlas*, to allow scientists to see which genes of the red flour beetle, *Tribolium castaneum*, are expressed in different tissues and at different stages of development. There are approximately 16,000 genes in the *Tribolium* genome, so that the structural description of many of them is based on computer prediction rather than experimental evidence. Separate initiatives to annotate the genome using different computer programs resulted in two sets of ‘gene models’: NCBI and OGS3. The latter predominates in the literature and was used in *BeetleAtlas*. The sequencing and the assembly of the sequenced fragments of the *Tribolium* genome is a continuing process, and a recent update was accompanied by a new NCBI gene set. In attempting to co-ordinate that with the OGS3 gene set we discovered major discrepancies, but neither gene set could be regarded as superior. To address this problem, we have produced *BeetleAtlas 2* (beetleatlas.org) as a bimodal application, allowing queries on instances of both gene models, but linked to a single visualization facility (a custom view in the UCSC Genome Browser) for comparison. This resolves many discrepancies and should prove useful in future refinement of the gene sets of this important model organism.

## Introduction

*Tribolium castaneum*, a member of the largest order of insects (*Coleoptera*), has emerged as a powerful model organism in insect functional genomics [1,2] where it is particularly suitable for studying developmental genetics [3], insect physiology [4–7] and population ecology [8]. Experimental work on *Tribolium* is facilitated by extensive genetic resources [9] and established CRISPR/Cas9 protocols [10]*.* In addition, a large-scale genome-wide RNAi screen has been performed on *Tribolium* [11] with the resulting data available from *iBeetle-Base* [12,13]*,* the *Tribolium* genomic web resource.

To aid studies of *Tribolium* [5] we constructed the web application, *BeetleAtlas* [14], which enables one to examine the expression of RNA transcripts of the individual genes of *Tribolium castaneum* in 22 different tissues of the adult and larva, and at different stages of embryonic development. This is reciprocally linked to *iBeetle-Base* where general information about the genes, their sequences, orthologues and RNAi phenotypes can be found.

The validity of the information in these resources depends upon the validity and accuracy of the identification of the genes themselves, which is a function of the accuracy of the assembled genome sequence and of its annotation. Like *iBeetle-Base*, the original *BeetleAtlas* used the then current version of the genome assembly — Tcas5.2 — and the annotation known as the ‘official gene set’, OGS3 [15], obtained using the prediction program, AUGUSTUS [16]. A different annotation was employed for the NCBI RefSeq gene set mounted on the NCBI/NLM website, obtained using a different gene-prediction program, Gnomon (https://www.ncbi.nlm.nih.gov/core/assets/genome/files/Gnomon-description.pdf). The identifiers for ‘gene models’ (annotated genes) from the OGS3 ‘gene set’ (complete genome annotation) are alphanumeric strings of the type ‘TC123456’, those from the NCBI gene set are six- or nine-digit numeric strings.

In late 2023 an improved assembly of the genome of *Tribolium castaneum* was released, icTriCast1.1, obtained from long-read sequencing with *PacBio Sequel* (https://www.ncbi.nlm.nih.gov/datasets/genome/GCF_031307605.1/). It was clearly necessary, therefore, to revise *BeetleAtlas*. The simplest course appeared to be to reprocess the RNA-seq data in relation to the new assembly and the associated gene set, and to establish equivalent pairs for OGS3 identifiers and the new NCBI identifiers so that users could search with the former, which predominate in the *Tribolium* literature. However, this course could not be adopted for two reasons: first, unambiguous equivalent pairs of gene identifiers could only be established in fewer than 60% cases and, second, the new NCBI gene set and the OGS3 set appeared to contain a similar proportion of questionably gene models. Therefore, a different approach was adopted for the new version of *BeetleAtlas* and is described here. This includes linked parallel ‘modes’ for the two gene models and provide a means of reconciling them (to the extent possible) through a customization of the UCSC Genome Browser for the icTriCast1.1 genome assembly.

## Design and implementation

### Data processing and database construction

An additional MySQL relational database for *BeetleAtlas 2* was populated with processed RNA-seq data as FPKM (Fragments Per Kilobase of transcript per Million mapped reads) generated using the Tuxedo pipeline [17] as previously [14], but with the icTriCast1.1 genome assembly and the NCBI annotations (https://genome.ucsc.edu/h/GCF_031307605.1) as reference. This was used for what we refer to as the ‘NCBI’ mode of the web application. The pre-existing database which used the Tcas5.2 genome assembly and the OGS3 annotations as reference was used for what we refer to as the ‘OGS3’ mode.

Both databases include RNA-seq data from two additional tissues — rectum and perirectal tubules — but did not include data for small RNAs as the 100 bp RNA-seq reads do not detect them. Data for mitochondrial transcripts were also excluded. The databases also include secondary data relating to *Drosophila* orthologues and *Tribolium* paralogues obtained variously using ‘Orthofinder’ [18], ‘EggNOG’ [19] and the Ensembl BioMart database at https://metazoa.ensembl.org/.

### Assigning equivalents between models the two gene sets

As indicated in the Introduction, the decision to redesign *BeetleAtlas* with two parallel modes was partly because unambiguous equivalent pairs of gene identifiers from the NCBI and OGS3 model sets could only be established in fewer than 60% cases. Nevertheless, it was desirable to include in *BeetleAtlas 2* those equivalent pairs that could be identified to allow cross-referencing between the two modes. A ‘minimal’ set of equivalents for gene models in the two sets was produced by BLAST [20] comparisons between predicted protein, mRNA and coding sequences (CDS) for the two models. Files OGS3_proteins.fasta, OGS3_mRNA.fasta and OGS3_CDS.fasta were downloaded from https://ibeetle-base.uni-goettingen.de/resources/. Files GCF_031307605.1_icTriCast1.1_protein.faa, GCF_031307605.1_icTriCast1.1_rna.fna and GCF_031307605.1_icTriCast1.1_cds_from_genomic.fna were downloaded from https://ftp.ncbi.nlm.nih.gov/genomes/all/GCF/031/307/605/GCF_031307605.1_icTriCast1.1/.

For each OGS3/NCBI pair, two standalone BLAST runs were performed, alternating query and database, with the parameters set so that only the single highest ‘hit’ was reported. A combined list of pairs of gene identifiers was generated, duplicates removed, and where multiple isoforms existed for the products of one gene, only the first was retained. This list was then processed to find any identifiers that appeared in more than one pair and these were removed. A final list of unique ‘equivalents’ was generated that included only the 8391 pairs that were present in all three lists—57% of protein-coding genes in the NCBI annotation and 50% of those in the OGS3 annotation (S1 Text). This may exclude some genuine matches but we felt that this was better than including unreliable ‘equivalences’. For each of the databases, these equivalents were incorporated into a field in the Gene table.

### Web application: General design

**Fig 1:**
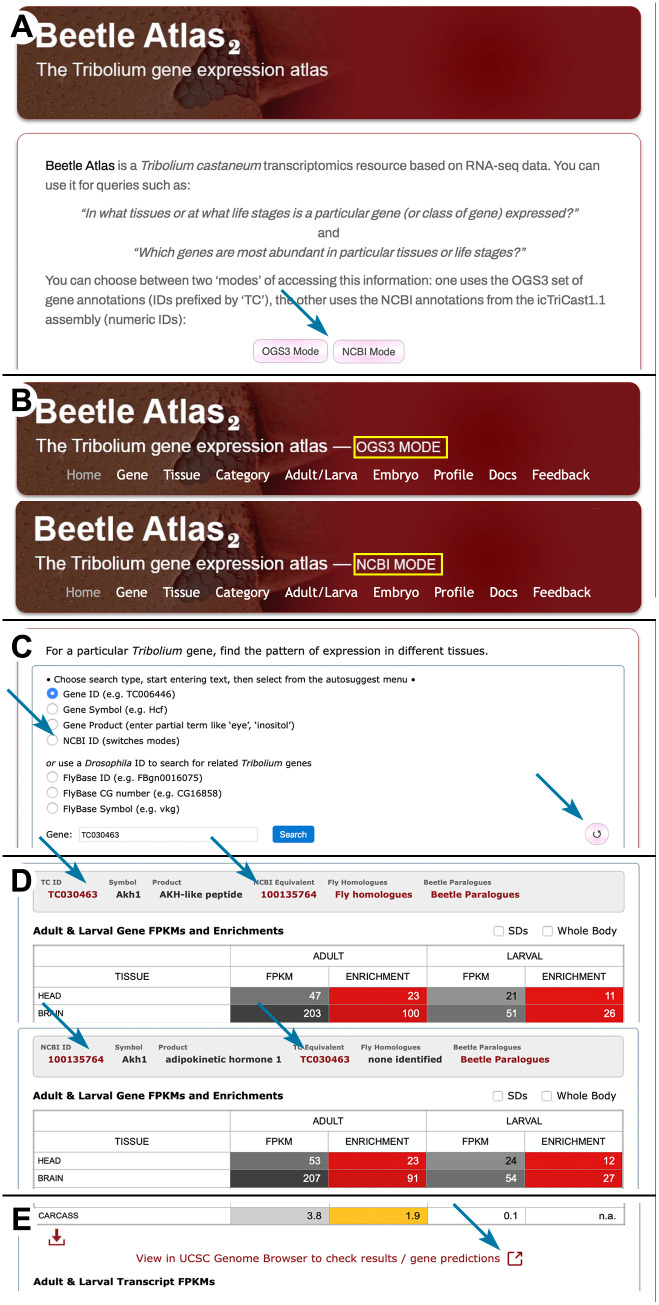
BeetleAtlas 2: New features. **(A)** Common entrance portal to two modes. **(B)** Differences in headers of two modes. **(C)** Gene search menu, showing mode-switch button. **(D)** Headers of Results tables in two modes. **(E)** Link out to UCSC Genome Browser view. Arrows indicate points referred to in the text.

The design of the *BeetleAtlas 2* web application incorporates what we term two ‘modes’ with web pages generated dynamically from separate Java Servlets and underlying MySQL databases running on Tomcat under Linux. A common entrance portal (Fig 1A) at the (redesignated) beetleatlas.org provides access to the two parallel modes.

The differences of the layouts of the two modes from that of the original *BeetleAtlas* (shown in Fig 1 of [14]) are as follows. Each web page includes a button that allows the user to switch from one mode to another (Fig 1C). There is an item for NCBI identifiers included in the input list in OGS3 mode, and vice versa, that initiates both switching and direct searching (Fig 1C). The summary header and associated links (Fig 1D) have been changed to include entries for both the gene identifier of the current mode (here OGS3) and any equivalent for the alternative mode that has been included in *BeetleAtlas 2*. Clicking on the gene identifier for the current mode links to the documentation of the gene model on either *iBeetle-Base* or the NCBI website, as appropriate, replacing previous separate links. Clicking on the ‘equivalent’ identifier does not switch modes, but brings up a new *BeetleAtlas 2* page for the gene model in the alternative mode. This allows side-by-side comparison of the tissue distribution of transcripts for the two modes. Although one might expect them always to be similar, the limitations of the method used to determine equivalences for *BeetleAtlas 2* means that this is not always the case, an example of which will be considered later. Links are provided between the gene and transcript results tables to the customized UCSC Genome Browser, described in the next section (Fig 1E).

### Integration with the UCSC genome browser

There are several genome browsers that have the facility of displaying a sequenced genome on which one may impose one’s own RNA-seq reads (in ‘bigWig’ format [21]) on parallel tracks [22–24]. We adopted the UCSC Genome Browser [22] as it supplied all the functionality that we required and we had used it previously with *FlyAtlas2* [25]. It presents parallel tracks of different features of the genome (e.g., GC percentage, repeat sequences, restriction enzyme sites), together with a track of the gene models of the NCBI RefSeq annotated genome. Most important, one can also include additional custom tracks, such as those for the RNA-seq read coverage in different tissues or alternative gene annotations. To integrate this with *BeetleAtlas 2* two types of custom tracks were required: tissue tracks and tracks of different gene sets, in each case related to the icTriCast1.1 genome assembly. This was to allow users to determine for themselves how the RNA-seq reads relate to particular gene models.

The tissue-specific transcript tracks were generated from ‘BAM’ files which are an intermediary in processing the raw RNA-seq reads through the Tuxedo pipeline. Replicate BAM files were merged, down-sampled to approximately the same size, and processed with the program, bam_to_bigwig [26], to generate files in the ‘bigWig’ format which is used for graphical display in the UCSC Genome Browser. Although the bigWig files related the RNA-seq reads to positions in the icTriCast1.1 assembly, their generation did not utilise the associated gene sets. Hence they can be used to compare the NCBI and OGS3 gene models imposed on this genome without prejudice, as illustrated in the Results section.

The UCSC Genome Browser provides a view of the icTriCast1.1 genome with an annotation track corresponding to the NCBI gene set, but can also display additional tracks of custom gene sets. Such tracks are generated from files in the ‘bigBed’ format, and are prepared from gtf (general transfer format) files following the procedure at UCSC (https://genome.ucsc.edu/goldenPath/help/bigGenePred.html). We prepared three custom tracks.

First, to allow comparison with the NCBI gene models, a custom track was generated for the OGS3 gene set. This was necessary as the sequence coordinates of the gene models in the OGS3 gene set relate to the Tcas5.2 genome assembly and cannot be used directly for display in the icTriCast1.1 view. We transferred the coordinates of the OGS3 gene set from the Tcas5.2 assembly (GCF_000002335.3) to the icTriCast1.1 assembly (GCF_031307605.1) using the program *Liftoff* [27]. This produced a gff (general file format) file containing 16,404 of the original 16,593 genes, and the file was converted to gtf format with the program, ‘gffread’ [28] from which a bigBed file was generated. The coordinates corresponding to the gene models in the converted gff file were uploaded to the *BeetleAtlas 2* database underlying the OGS3 mode to allow linkage to the UCSC browser view of icTriCast1.1.

Second, again using *Liftoff*, a custom track was generated for a subset of the NCBI gene set from the Tcas5.2 assembly (GCF_000002335.3) comprising gene models that had been discontinued in the icTriCast1.1 annotation. This track contained 1050 gene models from the 1102 that had been discontinued (S1 Text). It was included because in several instances the tissue transcripts appeared to support these earlier gene models rather than models in the new gene set, and because such discontinued identifiers may still be found in the literature. Gene data for these discontinued gene models were added to the database underlying the NCBI mode of *BeetleAtlas 2*.

Finally, although the UCSC Genome Browser display of the icTriCast1.1 assembly provides tracks for the NCBI Ref Seq annotation, we modified some of the nomenclature columns in the gtf file derived from the GCF_031307605.1 assembly and processed it to produce a track in which the gene models display the same gene ID or symbol that the user has been working with in *BeetleAtlas 2*, rather than the default transcript ID.

As already mentioned, the standard results page of a search in *BeetleAtlas 2* includes a link to a custom view in the UCSC Genome Browser (Fig 1E). This linkage is effected by a call to the UCSC Genome Browser specifying the genome position required and the location of the bigWig and bigBed files on the *BeetleAtlas 2* server. These are then retrieved and integrated into the display according to an associated text file.

### Presentation in the UCSC genome browser

**Fig 2:**
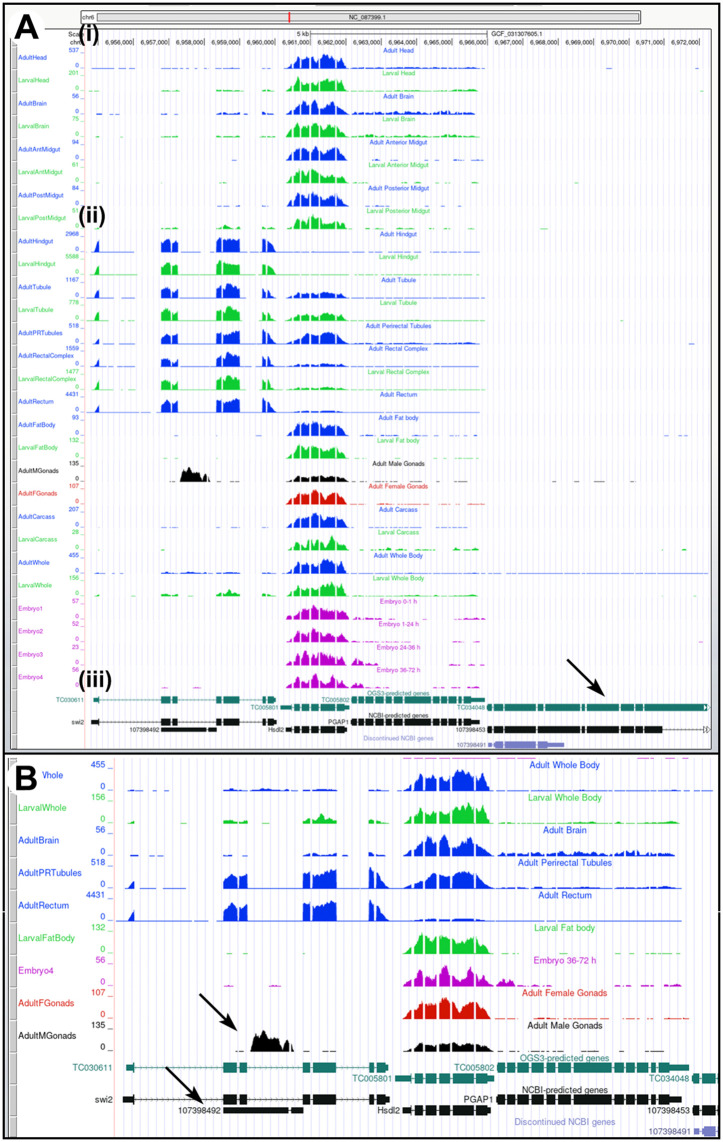
Customized UCSC Genome Browser view of transcript expression in region of a particular gene. **(A)** View of whole page, with chromosome representation **(i)**, custom tissue tracks (ii) and gene sets **(iii)**. A difference in the exons of two gene models is indicated by an arrow. **(B)** View of section of above after zooming in and reorganizing tracks. A gene model predicted in only one of the two gene sets and supporting transcript coverage is indicated by arrows.

An illustrative custom view of transcript data from *BeetleAtlas 2* in the UCSC Genome Browser is presented in Fig 2A. There are three sections—a representation of the chromosome with the ‘current’ position marked (i), tissue tracks (ii) and tracks for reference gene sets (iii). The default reference tracks provided by UCSC have been suppressed programmatically for clarity, but the interface provides controls for the user to show all or any of interest. In this respect, tracks of repeated sequences may be of importance in areas where there is signal but no gene model (S1 Text).

The use of these custom views is considered in detail in the Results section where the focus is on cases of discrepancy between OGS3 and NCBI gene models. However, the example shown illustrates the general similarity of gene models in many cases (albeit with minor differences—arrow) and the support provided by the tissue transcript tracks. For certain gene models signal is lacking and it is not possible to say whether the gene model is incorrect or whether the gene is not expressed in the conditions represented in *BeetleAtlas 2*.

The complexity of a view with tracks for 22 tissues is apparent, but tracks can be reorganized to bring those of most interest adjacent to the reference gene sets. In Fig 2B a signal found only in a single tissue (male gonads) has been moved to a position where it can be compared with gene model NCBI 107398492 and TC030611/swi2 in an intron of which it is located (arrows).

## Results

### Examining gene models in the UCSC genome browser: Strategy

**Fig 3:**
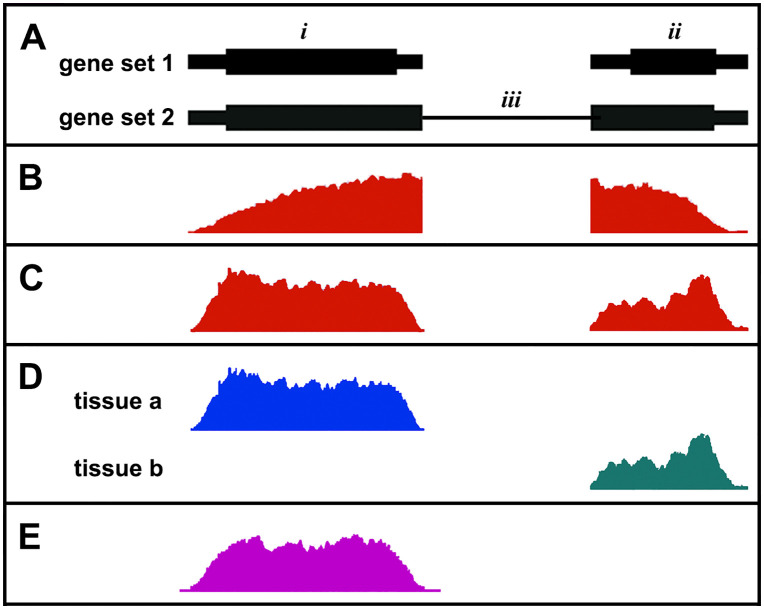
Distinguishing between two gene models. **(A)** Simplified diagrams of two gene models. Introns in i and ii have been omitted for clarity. **(B)** RNA-seq reads for a single tissue in which two regions have an abrupt discontinuation between them. **(C)** RNA-seq reads for a single tissue in which there are two separate regions, each showing a gradual decline at the 5′ and 3′ ends. **(D)** RNA-seq reads for a two tissues, showing coverage in alternative sub-regions. **(E)** RNA-seq reads for a single tissue in which there is coverage of only one of the sub-regions.

The customized UCSC Genome Browser can be used to validate, compare and resolve gene models from the two gene sets. For those readers unfamiliar with it, we illustrate the general approach in relation to conflicting gene models in a hypothetical region of the genome (Fig 3). It is frequently found that one gene set presents two adjacent gene models (*i* and *ii* in Fig 3A, lacking introns for clarity), whereas the other presents a single gene model in which two exons (or groups of exons) are linked by an intron (*iii* in Fig 3A). Assuming that there is significant expression in at least some tissues (without which nothing can be concluded), three main situations are of interest.

Fig 3B shows the situation where transcripts map at a similar level to both sub-regions. One interpretation of this is that there is a single gene (represented by model iii) in this region. A less likely possibility is that there are two separate genes expressed to a similar extent in the tissue.

Such alternatives can often be resolved by considering the appearance of the RNA-seq tracks. It is found that at the junction of an intron and an exon there is a sharp discontinuity in the RNA reads (a ‘cliff-edge’), whereas towards the 5′ and 3′ ends of a mRNA the coverage shows a gradual decrease in amplitude. (This latter is caused by a variety of factors, including under-representation of fragments from the ends and loss during size-selection because there is a greater proportion of smaller fragments.) The ‘cliff-edge’ provides a particularly clear diagnostic. It should be remarked that the RNA-seq coverage of mRNAs is never uniform, and gradual decline is sometimes observed at points within an exon. Such cases are generally easy to distinguish from separate genes as there tends to be no discrete separation.

Returning to Fig 3B, it can be seen that the ‘cliff-edges’ are consistent with the presence of an intron in the DNA (model *iii*). In contrast, Fig 3C shows the gradual decline expected for two distinct genes. An indication of two distinct genes (represented by models *i* and *ii)* is very strong in Fig 3D, where transcripts map to only one sub-region in ‘tissue a’ and only to the other in ‘tissue b’. An alternative explanation in terms of differential splicing resulting in tissue-specific transcripts would require a gene model in which the proteins resulting from these transcripts shared no common regions. This would not conform to the concept that differentially spliced transcripts of the pre-mRNA of a single gene encode proteins that, although differing from one another, share some common functionality. Moreover we have not encountered such gene models in either of the gene sets in *Tribolium*. Nevertheless it should be stated that 38 such examples have been found in *Drosophila melanogaster*, representing about 0.2% of all genes [29].

Fig 3E show a related situation to those in Fig 3C and 3D, but in which transcripts map to only one sub-region. This would appear to be evidence for one of the gene-models (here *i*) in set 1, which predicts two genes. However, no conclusion can be made regarding gene model *ii*. Furthermore, even if it has not been included in a particular annotation, it is possible that *iii* could represent an alternative transcript to *i*, and might be found in another tissue or at a different life stage.

### Examining gene models in the UCSC genome browser: Examples

**Fig 4:**
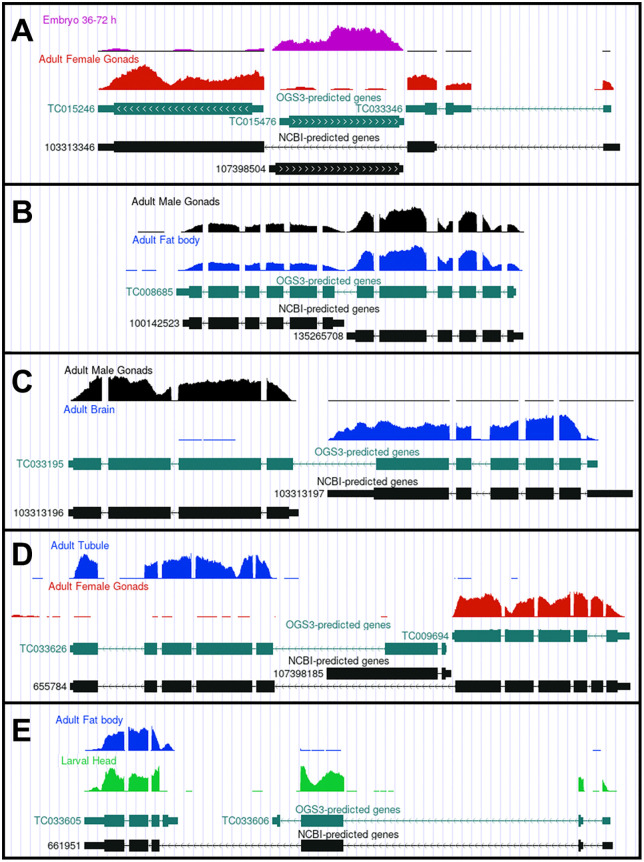
Examples of using the UCSC Genome Browser to compare gene models. The examples were all generated by a search for a particular gene in *BeetleAtlas 2*, followed by linking out to the custom view in the UCSC Genome Browser. The regions of the icTriCast1.1 genome assembly involved are **(A)** Chromosome 6: 4,841,597–4,847,402. **(B)** Chromosome 3: 10,819,898–10,824,887. **(C)** Chromosome 5: 3,661,574–3,669,062. **(D)** Chromosome 3: 20,097,118–20,110,251. **(E)** Chromosome 3: 20,839,118–20,854,932.

Fig 3 presents simple hypothetical cases, explaining the approach used. Actual, more complex, examples are shown in Fig 4. Fig 4A (an example of Fig 3B) shows a sharp interruption between the two regions of expression in female gonads, consistent with the single-gene prediction (103313346) of the NCBI gene set (albeit with an extra exon), rather than the two-gene prediction of the OGS3 gene set (TC015246 and TC033346). The situation is slightly complicated by a single-exon gene (predicted by both gene sets) within the intron.

Fig 4B illustrates an example of Fig 3C, in which the NCBI two-gene prediction is favoured over the OGS3 one-gene prediction because of the gradual trail-off of signal at the ends of the two regions.

The less equivocal situation of Fig 3D is illustrated in Fig 4C, where independent expression of the two regions in male gonads and adult brain provides strong support for the two-gene prediction. (However, the 5′ untranslated region predicted for 103313197 may be too long.)

There are also cases where more substantial differences from both models are suggested by examining the transcript reads. Fig 4D shows a region in which the OGS3 gene set had two gene models (TC033626 and TC009694), whereas the NCBI gene set has only one (655784). The NCBI gene model 655784 is clearly inconsistent with the different genomic regions of expression in female gonads and adult tubules. However, while the pattern of expression in female gonads provides general support to the assignation TC009694, the RNA-seq reads indicate an additional exon (2), absent from this gene model. Furthermore, although the pattern of expression in adult tubule is consistent with five exons of TC033626, the first two predicted exons are not covered by RNA-seq reads. Of course, it cannot be excluded that TC033626 and TC009694 have alternative transcripts to those predicted in their respective gene models.

Fig 4E shows a situation where the differences between gene models in the two sets could well arise from differential transcripts originating from the RNA precursor of a single gene. The single transcript predicted by the NCBI gene model (661951) is strongly supported by the distribution of reads in larval head. However, although this would appear to exclude one of the OGS3 models (TC033606), the other (TC033605) is generally consistent with the pattern of expression in adult fat body. One possibility is that TC033605 represents a transcript with the last three exons of 661951 together with an additional exon. However, in view of the absence of a ‘cliff edge’ at the division with the putative additional exons 1, the model for TC033605 may be incorrect, and the transcript encompass the last two exons of 661951 together with an extended preceding exon.

### Examining gene models in the UCSC genome browser: Use cases

The preceding sections explain the principles of interpretating RNA-seq reads in the UCSC Genome Browser using examples encountered after linking out from results sets in *BeetleAtlas 2*. We now consider systematically how the web application is used in the different circumstances confronting experimental scientists (Table 1) to help them employ it most effectively and make appropriate interpretation of their results. In addition this will demonstrate the deficiencies of both gene sets that necessitated a bimodal structure for *BeetleAtlas 2*.

**Table 1:** BeetleAtlas 2/UCSC Genome Browser Use Cases.

Situation	BeetleAtlas 2		UCSC Analysis of RNA-seq read coverage	Inference	Example
**Signal**	**Comparison**
Equivalents in BeetleAtlas 2	No	n/a	No coverage	None possible	—
			Coverage	Error	—
	Yes	Similar	Models similar/Coverage fits models	Confirms models and equivalence	—
			Models similar/Coverage does not fit	Error in one or both models	Fig 5A
	Yes	Differ	Models differ/Coverage does not fit	Error in one or both models	Fig 5B
No Equivalents in BeetleAtlas 2	No	—	No coverage	None possible	—
			Coverage	Error	—
	Yes	—	Coverage fits model 1/No model 2	Confirms model 1/model 2 deficiency	Fig 5C
			Coverage fits both models 1 and 2	Equivalence/Models confirmed or adjusted	Fig 5D
			Coverage fits one model but not other	Confirms one model and negates other	Fig 4
Discontinued NCBI ID	n/a	—	No coverage	None possible	—
			Coverage fits revised NCBI model	Removal correct	—
			Coverage fits original NCBI model	Removal incorrect	Fig 5D
			Coverage fits neither NCBI model	Model correction required	—
Undocumented UCSC coverage	n/a	—	Signal in area with no model gene	Possible new gene	Fig 5E

**Fig 5:**
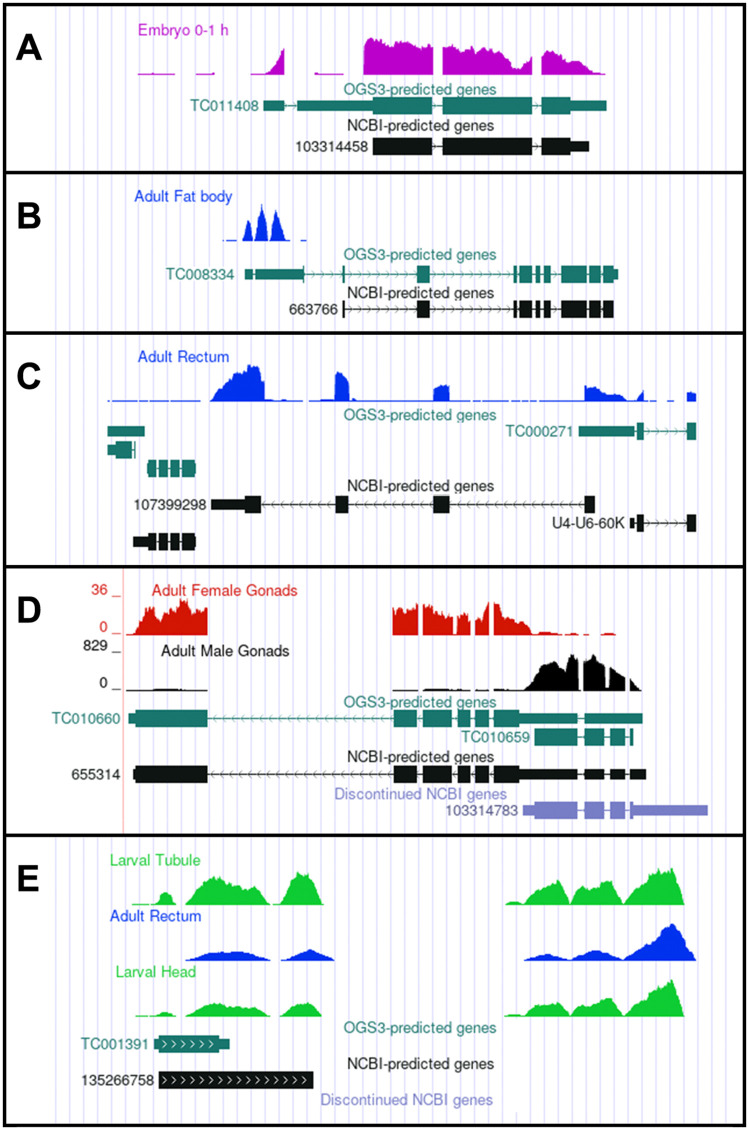
BeetleAtlas 2/UCSC Genome Browser — illustration of use cases. Custom views in the UCSC Genome Browser linked from *Beetle Atlas 2* in different situations. **(A)** Link out from TC011408 or 103314458 reveals discrepancy in gene models reported as equivalent and showing similar expression of transcripts across tissues. Gene model 103314458 appears to be incorrectly lacking an exon. **(B)** Link out from TC0008334 or 663766 explains the different reported expression of transcripts across tissues in gene models reported as equivalent. Gene model TC0008334 appears incorrect and the two designated 5′ exons appear to represent a separate gene. **(C)** Link out from 107399298, with no reported equivalent, indicates that this corresponds to a gene missing from the OGS3 gene set. **(D)** Link out from TC010660 or 655314 indicates equivalence, despite not being reported as such, because of overlap of gene models with TC010659. Discontinued 103314783 also shown. **(E)** Link out from TC001391 or 135266758 reveals adjacent region of the genome with coverage by RNA-seq reads but lacking a corresponding gene model.

First, we shall consider the instances (8391 in total) in which *BeetleAtlas 2* reports an equivalent to a gene of interest in the mode complementary to the one being used. Assuming that the gene is expressed in at least one tissue or life stage, the user should invoke the alternative mode (by clicking on the corresponding identifier) to check whether the tissue distribution is similar in the two cases. Where this is the case, the only reason to invoke the UCSC Genome Browser would perhaps be to see if the RNA-seq reads completely supported the gene models. Generally this will be so, but occasional discrepancies are observed (S1 Text). However, invoking the custom UCSC Genome Browser view is clearly necessary where there are different patterns of tissue expression between reported ‘equivalents’. An example of this type (Fig 5B) involves expression restricted to two of eleven predicted exons which are only present in one of the gene models and seem to represent a distinct gene. Of course, for instances where there is no gene expression in the tissues or life stages represented, examination in the UCSC Genome Browser can do no more than check for rare errors in either the gene models or the processing of the data.

The next situation concerns the 7014 NCBI and 8013 OGS3 gene models for which *BeetleAtlas 2* reports no complementary equivalent. Many of these have transcripts in at least one tissue or condition, justifying attempts to identify equivalent gene models using the customized UCSC Genome Browser. Cases where there is a conflict between one or two predicted genes have already been considered (Figs 3 and 4). Other cases occur in which one gene set has no corresponding gene model in a region of RNA-seq reads (e.g., Fig 5C). There are also cases where an equivalence exists, but has been missed, often because of overlapping genes. Fig 5D shows that the gene models TC010660 and 655314 are very similar except for the presence or absence of two introns at the 5′ end of the putative transcript. However, the latter overlaps TC010659, explaining why the equivalence was missed as ‘not unique’. This particular example is of further interest. The results tables in *BeetleAtlas 2* present high expression in adult male gonads (and adult fat body) for all three gene models. However, whereas TC010660 and 655314 show a low background of expression in other tissues, including adult female gonads, TC010659 does not (S1 Text). It is evident from Fig 5D that in TC010660 and 655314 there are no RNA-seq reads corresponding to exons other than the first two in male gonads. Furthermore, the RNA-seq reads are consistent with four exons (as in TC010659), rather than the two predicted. Hence the tables presented in *BeetleAtlas 2* for these gene models are almost certainly incorrect. This highlights the fact that the validity of results in *BeetleAtlas* tables depend on the validity of the gene models used to obtain them. Moreover the ‘equivalence’ of gene models does not necessarily mean that they are correct.

Fig 5D also illustrates that the original NCBI gene set included a now discontinued gene model (103314783) that corresponds, albeit imperfectly, to TC010659. The inclusion of the discontinued NCBI gene models in the database underlying *BeetleAtlas 2* allows direct queries of such gene models to enable examination using the customized UCSC Genome Browser view. In many cases the RNA-seq coverage supports the new model. Nevertheless, in other instances — as in Fig 5D — the RNA-seq coverage fits better with the previous one.

The final illustration is of transcript coverage for a region of the genome for which neither NCBI nor OGS3 gene models exist, suggesting a previously unannotated gene or genetic element. This example — a chance observation — is shown on the right of Fig 5E. Further investigation revealed that this region of the genome contains a retrotransposon (S1 Text). On the basis of sequence analysis and comparison this appears to be a class I LTR retrotransposon belonging to the *Gypsy* superfamily, and we have given it the name, *Pucelle*. It is quite distinct from another previously reported *Tribolium* transposon of the same class, *Woot* [30].

## Availability and future directions

### Availability

*BeetleAtlas 2* is freely available for use without registration at www.beetleatlas.org. Extensive online documentation is provided, and a PDF manual is available for download. RNA-Seq data have been deposited with the European Nucleotide Archive under accession number PRJEB110472. The code for the Java Servlets is available at https://github.com/AonachMor/BeetleAtlas.

### Limitations and constraints

*BeetleAtlas 2* does not provide reliable results for genes such as those for histones that are present in multiple identical or near identical copies. For such genes false negatives or positives occur for either or both of the tabulated tissue-specific expression or the graphed read coverage. This arises from the way the software used to process the RNA-seq files attempts to assign and quantify transcription in such cases, and is a common problem in resources of this type, e.g., *FlyBase* [31]. *BeetleAtlas 2* informs the user of this problem for genes with paralogues of 99% or greater nucleic acid sequence identity.

### Future directions

The custom UCSC Genome Browser view significantly enhances *BeetleAtlas* by allowing users to check the validity of tabulated results for tissue- or stage-specific expression of a *Tribolium* gene of interest — the purpose for which it was designed. However, additional possibilities have emerged from this. It is evident that despite the progress in sequencing and annotating the *Tribolium* genome, both the OGS3 and the current gene NCBI gene sets still have significant shortcomings and a revised genome annotation is clearly necessary. In this respect the UCSC Genome Brower view of the tissue transcripts in relation to equivalent OGS3 and NCBI gene models can resolve discrepancies between them and could, therefore, play an important part in a revised annotation.

If employed in such a role it might be advantageous to extend *BeetleAtlas 2* so that automated new or revised annotations could be generated from the genome browser. The most commonly used editing platform for genome annotation is *Web Apollo* [32] which is integrated into a different genome browser — *JBrowse* [23]. The tabular presentation of quantitative transcriptomic data in *BeetleAtlas 2* is separate from the visual presentation of the RNA-seq reads in the UCSC genome browser. Thus, the links to the remote UCSC Genome Browser could easily be replaced by those to a local installation of *JBrowse*, which accepts the same bigWig- and bigBed-format files.

Until such a time as a new annotation initiative is undertaken for the *Tribolium* genome, we plan to use *BeetleAtlas 2* to make publicly available the additions and corrections that accumulate. We intend to construct and continually update two gff files and use them to generate additional custom tracks for the UCSC Genome Browser view. One of these tracks will be for transposable elements, which are not currently included in the NCBI gene set for the icTricast1.1 genome assembly. We have already implemented this, although at the time of writing it is only populated with the elements of the family *Pucelle*, described here. The other track will be for corrections and additions involving protein-coding genes. This will allow users of *BeetleAtlas 2* to be informed of any change in status of their genes of interest, and provide an archive in standard format for use in the future. We encourage other workers in the *Tribolium* field to contribute any corrections or additions they find for inclusion in updates of the web application.

## Supporting information

S1 Text Supplementary Information for the paper. (PDF)
