# Supplementary material for: BeetleAtlas 2: An enhanced Tribolium castaneum web resource for tissue and developmental transcriptomics allowing refinement of gene predictions
Source: PLoS Comput Biol. 2026 Jun 1;22(6):e1014314. doi: 10.1371/journal.pcbi.1014314 (PMC13245856; doi:10.1371/journal.pcbi.1014314)
Supplement: S1 Text — (PDF) [file pcbi.1014314.s001.pdf]

SUPPLEMENTARY INFORMATION

FOR THE PAPER

BeetleAtlas 2: An enhanced *Tribolium castaneum* web resource for tissue and developmental transcriptomics allowing refinement of gene predictions

David P. Leader[1], Muhammad T. Naseem[2], Janina Rinke[3], and Kenneth V. Halberg[2]

[1]Institute of Molecular Cell and Systems Biology, College of Medical, Veterinary and Life Sciences, University of Glasgow, Glasgow G12 8QQ, UK

[2]Department of Biology, Section for Cell and Neurobiology, University of Copenhagen, Copenhagen DK-2100, Denmark

[3]Institute for Evolution and Biodiversity, University of Münster, 48149 Münster, Germany

Table of Contents

1. Unique equivalents between OGS3 and NCBI gene models 3

2. NCBI gene models discontinued in the icTriCast1.1 annotation 4

3. Example of repeat region producing signal in absence of gene model 7

4. Similarities and differences of gene models in the icTriCast1.1 assembly 8

5. Similar expression of equivalent gene models in the two gene sets......................... 9

6. Different expression of 'equivalent' gene models in the two gene sets 10

7. Dubious reporting of tissue expression because of suspect gene models 11

8. Putative retrotransposon, *Pucelle* 12

1. Unique Equivalents between OGS3 and NCBI Gene Models

A. OGS3 and NCBI Genes with one or more matches to other Gene Set

Annotation	Total Genes	Match	No Matches
OGS3	16,593	13,212	3381
NCBI	14,838	12,089	2149

Matches were on the basis of protein–protein comparisons. The discrepancy between the number of matches is explained by situations such as those in which a gene model in Set A has matches to two models in Set B, but these are the single matches for the models in Set B.

B. Unique gene pairs from different BLAST runs on OGS3 and NCBI gene sets

Protein	mRNA	CDS	Common
9409	8806	9587	**8319**

Protein–Protein, RNA–RNA and CDS–CDS BLAST runs were performed and the total matches were refined to give those unique matches in which each gene model had only a single ‘equivalent’. Further refinement gave the 8319 instances which were common to each run. These are described as “unambiguous” in the main text.

2. NCBI gene models discontinued in the icTriCast1.1 annotation

The following lists the 1050 discontinued NCBI gene models used to populate the custom track for the UCSC Genome Browser view of *BeetleAtlas 2*.

100141530	103312336	103312773	103313287	103313745	103314175
100141531	103312344	103312782	103313293	103313747	103314179
100141598	103312351	103312784	103313298	103313759	103314185
100141699	103312353	103312796	103313312	103313763	103314186
100141732	103312365	103312798	103313316	103313778	103314190
100141759	103312380	103312807	103313330	103313783	103314207
100141819	103312384	103312814	103313331	103313793	103314208
100141826	103312390	103312816	103313348	103313797	103314230
100141835	103312392	103312823	103313349	103313803	103314231
100141874	103312394	103312829	103313356	103313804	103314233
100141893	103312397	103312837	103313359	103313805	103314236
100141906	103312400	103312838	103313375	103313812	103314263
100142014	103312403	103312859	103313381	103313814	103314272
100142017	103312404	103312863	103313386	103313820	103314283
100142031	103312413	103312905	103313393	103313824	103314291
100142037	103312416	103312916	103313403	103313829	103314303
100142043	103312421	103312931	103313404	103313836	103314306
100142066	103312422	103312938	103313406	103313839	103314312
100142122	103312433	103312941	103313416	103313853	103314315
100142148	103312437	103312943	103313428	103313857	103314320
100142239	103312443	103312950	103313433	103313863	103314336
100142255	103312471	103312964	103313437	103313867	103314338
100142256	103312473	103312965	103313452	103313869	103314351
100142308	103312475	103312967	103313459	103313878	103314352
100142335	103312476	103312983	103313462	103313881	103314357
100142345	103312486	103312988	103313465	103313884	103314363
100142352	103312487	103312991	103313467	103313888	103314372
100142373	103312490	103312997	103313470	103313894	103314380
100142396	103312512	103313012	103313472	103313896	103314383
100142407	103312524	103313025	103313473	103313906	103314384
100142427	103312537	103313037	103313478	103313911	103314391
100142439	103312540	103313045	103313485	103313915	103314401
100142502	103312556	103313058	103313495	103313921	103314409
100142583	103312557	103313069	103313504	103313938	103314410
100142588	103312566	103313078	103313506	103313943	103314421
100142622	103312576	103313089	103313512	103313948	103314429
100142624	103312577	103313109	103313517	103313950	103314430
100462677	103312581	103313121	103313523	103313956	103314434
103312117	103312590	103313126	103313529	103313960	103314437
103312140	103312594	103313128	103313536	103313963	103314449
103312147	103312598	103313135	103313584	103313964	103314454
103312148	103312601	103313146	103313591	103313968	103314456
103312151	103312602	103313147	103313596	103313981	103314461
103312153	103312611	103313150	103313599	103313987	103314468
103312154	103312613	103313151	103313602	103313995	103314473
103312156	103312615	103313167	103313613	103314001	103314475
103312174	103312618	103313168	103313615	103314018	103314501
103312233	103312623	103313172	103313616	103314019	103314502
103312240	103312626	103313173	103313631	103314026	103314537
103312242	103312635	103313201	103313652	103314035	103314538
103312243	103312637	103313205	103313658	103314039	103314554
103312258	103312657	103313210	103313659	103314046	103314557
103312266	103312664	103313213	103313664	103314064	103314568
103312280	103312669	103313214	103313672	103314070	103314570
103312281	103312673	103313215	103313679	103314072	103314571
103312282	103312688	103313221	103313683	103314086	103314591
103312286	103312692	103313222	103313686	103314124	103314595
103312291	103312703	103313225	103313698	103314127	103314600
103312300	103312713	103313235	103313711	103314134	103314602
103312305	103312723	103313252	103313723	103314141	103314603
103312320	103312751	103313259	103313728	103314155	103314613
103312326	103312758	103313265	103313736	103314169	103314614
103312327	103312760	103313282	103313738	103314174	103314623

103314632	103315093	107397667	107397988	107398294	107398614
103314635	103315109	107397668	107397999	107398295	107398633
103314636	103315113	107397669	107398002	107398314	107398641
103314637	103315118	107397670	107398003	107398325	107398643
103314646	103315122	107397671	107398031	107398328	107398645
103314653	103315123	107397673	107398032	107398330	107398658
103314665	103315132	107397676	107398034	107398331	107398659
103314666	103315171	107397677	107398035	107398334	107398664
103314667	103315174	107397678	107398038	107398338	107398667
103314674	103315176	107397680	107398041	107398339	107398684
103314677	103315184	107397685	107398042	107398341	107398687
103314682	103315188	107397687	107398043	107398348	107398695
103314683	103315195	107397688	107398044	107398357	107398696
103314697	103315198	107397694	107398049	107398359	107398702
103314700	103315201	107397695	107398050	107398360	107398712
103314715	103315252	107397710	107398051	107398363	107398714
103314742	103315261	107397711	107398056	107398364	107398720
103314743	107397395	107397712	107398061	107398365	107398727
103314746	107397401	107397713	107398068	107398366	107398729
103314749	107397402	107397717	107398069	107398376	107398730
103314763	107397407	107397718	107398075	107398377	107398735
103314764	107397413	107397722	107398076	107398388	107398736
103314765	107397422	107397725	107398081	107398390	107398738
103314766	107397426	107397729	107398082	107398395	107398739
103314783	107397427	107397731	107398084	107398396	107398740
103314788	107397431	107397732	107398087	107398406	107398741
103314797	107397440	107397743	107398092	107398407	107398746
103314808	107397443	107397749	107398093	107398412	107398760
103314835	107397444	107397761	107398095	107398413	107398766
103314842	107397445	107397766	107398096	107398414	107398768
103314869	107397446	107397767	107398098	107398415	107398779
103314871	107397457	107397771	107398100	107398416	107398781
103314889	107397460	107397776	107398101	107398425	107398783
103314892	107397469	107397793	107398117	107398440	107398784
103314902	107397475	107397794	107398129	107398449	107398785
103314908	107397484	107397795	107398132	107398454	107398786
103314909	107397488	107397800	107398133	107398456	107398787
103314910	107397490	107397801	107398159	107398458	107398788
103314911	107397491	107397807	107398164	107398469	107398791
103314919	107397493	107397809	107398166	107398471	107398792
103314920	107397494	107397820	107398172	107398476	107398797
103314922	107397499	107397823	107398186	107398491	107398798
103314925	107397511	107397824	107398200	107398495	107398800
103314936	107397518	107397825	107398201	107398501	107398802
103314943	107397519	107397827	107398202	107398503	107398805
103314952	107397535	107397834	107398207	107398507	107398813
103314965	107397537	107397839	107398208	107398509	107398814
103314967	107397538	107397864	107398214	107398518	107398816
103314977	107397558	107397870	107398215	107398524	107398821
103314985	107397574	107397879	107398216	107398527	107398822
103314992	107397580	107397886	107398217	107398529	107398834
103314997	107397585	107397888	107398218	107398530	107398836
103315001	107397590	107397899	107398219	107398532	107398839
103315020	107397591	107397907	107398220	107398535	107398840
103315021	107397596	107397931	107398221	107398547	107398841
103315024	107397602	107397934	107398222	107398558	107398842
103315042	107397608	107397935	107398224	107398562	107398854
103315049	107397609	107397938	107398227	107398564	107398857
103315061	107397610	107397941	107398230	107398565	107398858
103315063	107397614	107397952	107398234	107398566	107398866
103315064	107397615	107397953	107398237	107398570	107398868
103315066	107397616	107397962	107398238	107398571	107398871
103315068	107397619	107397971	107398250	107398593	107398877
103315069	107397625	107397972	107398256	107398594	107398878
103315070	107397639	107397973	107398257	107398599	107398880
103315074	107397646	107397982	107398262	107398600	107398883
103315075	107397657	107397983	107398284	107398604	107398885
103315076	107397666	107397985	107398289	107398608	107398888

107398893	107399037	107399195	656136	659285	661966
107398896	107399040	107399197	656206	659364	661977
107398901	107399041	107399200	656232	659402	661983
107398905	107399050	107399203	656254	659416	662004
107398907	107399051	107399216	656392	659439	662037
107398908	107399056	107399217	656487	659441	662142
107398909	107399060	107399225	656546	659470	662151
107398910	107399063	107399232	656585	659519	662286
107398911	107399065	107399239	656601	659538	662360
107398916	107399070	107399243	656629	659613	662392
107398921	107399073	107399248	656672	659620	662421
107398923	107399075	107399250	656824	659769	662466
107398924	107399077	107399252	656877	659792	662534
107398928	107399080	107399253	656963	659819	662699
107398929	107399091	107399255	657043	659913	662738
107398933	107399095	107399258	657130	660050	662758
107398935	107399096	107399265	657152	660080	662773
107398944	107399098	107399268	657191	660114	662801
107398949	107399100	107399269	657230	660123	662821
107398954	107399108	107399273	657277	660215	662879
107398957	107399111	107399280	657309	660429	662979
107398958	107399112	107399281	657430	660496	663018
107398959	107399113	107399293	657433	660577	663077
107398960	107399115	107399297	657514	660583	663085
107398961	107399117	654882	657516	660749	663114
107398969	107399121	654890	657579	660793	663115
107398970	107399125	654906	657600	660809	663143
107398972	107399126	654933	657684	660911	663145
107398973	107399130	655017	657836	661005	663189
107398974	107399132	655019	658008	661062	663326
107398977	107399134	655079	658009	661066	663335
107398979	107399135	655216	658062	661094	663455
107398980	107399136	655285	658086	661108	663482
107398984	107399137	655309	658091	661257	663506
107398987	107399138	655323	658175	661283	663581
107398989	107399139	655324	658253	661303	663710
107398990	107399141	655328	658285	661320	663762
107398997	107399147	655446	658325	661351	663770
107398999	107399150	655474	658387	661366	663839
107399000	107399152	655572	658413	661371	663925
107399001	107399154	655615	658446	661374	664052
107399003	107399158	655713	658465	661376	664189
107399005	107399159	655794	658492	661384	664192
107399006	107399164	655820	658549	661527	664318
107399007	107399165	655848	658680	661621	664331
107399008	107399177	655875	658891	661651	664410
107399013	107399180	655877	658961	661773	664412
107399015	107399181	655963	658998	661819	664431
107399017	107399183	655967	659002	661850	664475
107399018	107399187	655991	659019	661886	664569
107399024	107399188	656005	659159	661903	664589
107399030	107399190	656012	659213	661911	
107399032	107399193	656080	659270	661917	

3. Example of repeat region producing signal in absence of gene model

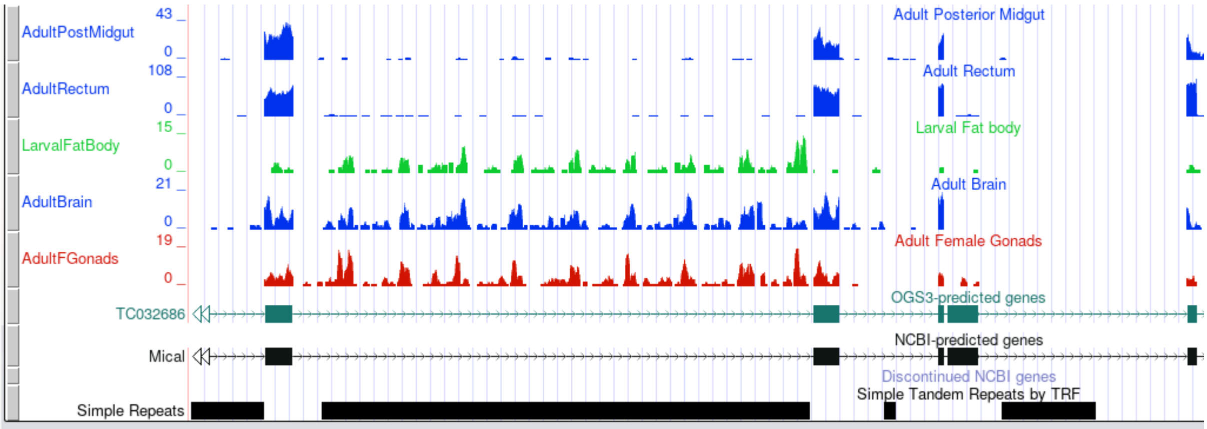

The illustration shows some of the tissue tracks in which a recurring signal within an intron of gene TC032685/Mical fall within a region of established Simple Repeats. (Some of the predicted alternative transcripts have been removed for clarity.)

4. Similarities and differences of gene models in the icTriCast1.1 assembly

A. Arbitrary section of the genome

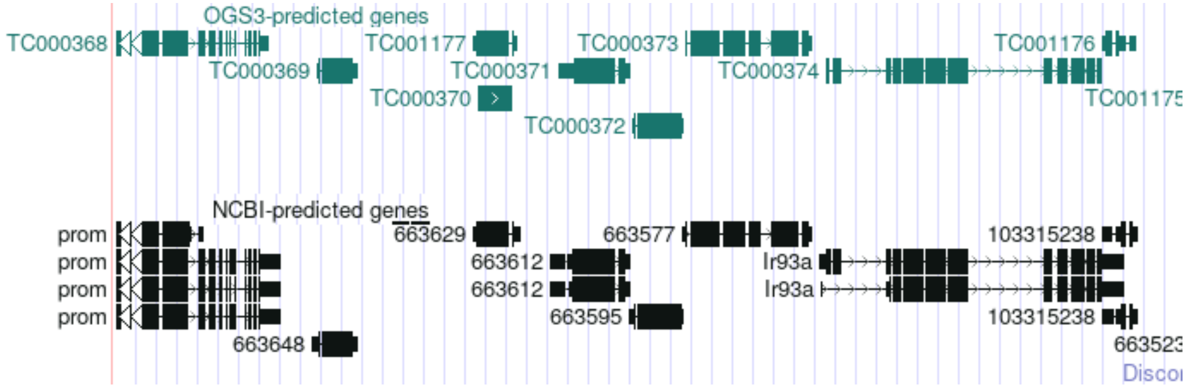

…continued

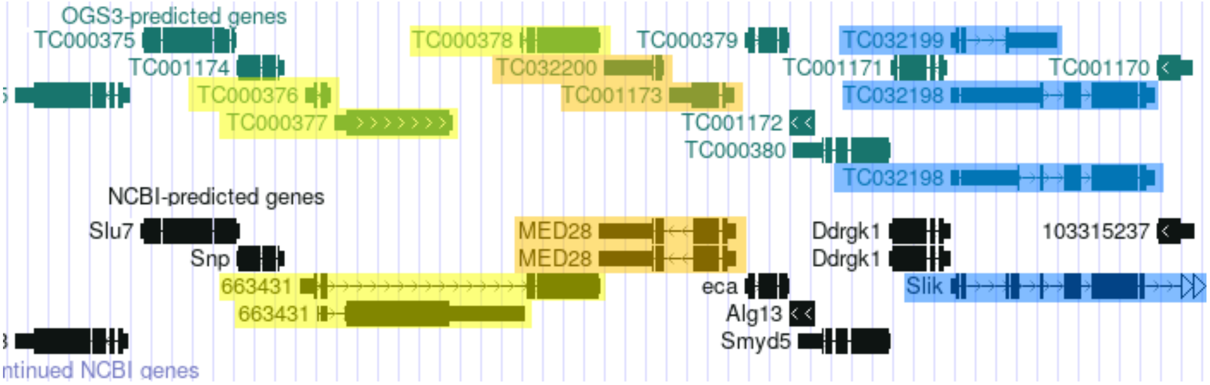

Eighteen gene models are shown. Differences are highlighted in similar colours.

B. Comparison of gene models in a data set representing a specific pattern of expression

**Both judged correct**	58	90	65%
**Minor differences**	32		
**Major differences †**	39	48	35%
**Both judged incorrect**	9		
**Total**	138		
† OGS3 judged correct	17		
† NCBI judged correct	22		

The dataset was for genes co-expressed exclusively in adult fat body and male gonads.

5. Similar expression of equivalent gene models in the two gene sets

A. TC011408 (OGS3)

TC ID	Symbol	NCBI Equivalent	Fly Homologues	Beetle Paralogues
TC011408	LOC103314458	103314458	none identified	none identified

Adult & Larval Gene FPKMs and Enrichments ☐ SDs ☐ Whole Body

	ADULT		LARVAL	
TISSUE	FPKM	ENRICHMENT	FPKM	ENRICHMENT
HEAD	6.0	1.3	7.9	1.1
BRAIN	3.6	0.8	5.0	0.7
ANTERIOR MIDGUT	9.4	2.0	5.7	0.8
POSTERIOR MIDGUT	9.5	2.1	4.9	0.7
HINDGUT	11	2.4	7.6	1.1
TUBULES (FREE)	4.5	1.0	4.6	0.7
PERIRECTAL TUBULES	8.0	1.7		
RECTUM	9.1	2.0		
RECTAL COMPLEX	7.8	1.7	8.0	1.1
FAT BODY	4.7	1.0	9.2	1.3
MALE GONADS	6.8	1.5		
FEMALE GONADS	24	5.1		
CARCASS	11	2.5	17	2.5

B. 103314458 (NCBI)

NCBI ID	Symbol	Product	TC Equivalent	Fly Homologues	Beetle Paralogues
103314458	LOC103314458	hypothetical protein	TC011408	none identified	none identified

Adult & Larval Gene FPKMs and Enrichments ☐ SDs ☐ Whole Body

	ADULT		LARVAL	
TISSUE	FPKM	ENRICHMENT	FPKM	ENRICHMENT
HEAD	8.4	1.3	9.1	1.0
BRAIN	5.6	0.9	7.9	0.9
ANTERIOR MIDGUT	13	2.0	7.9	0.9
POSTERIOR MIDGUT	14	2.1	7.4	0.8
HINDGUT	18	2.8	13	1.4
TUBULES (FREE)	6.4	1.0	6.6	0.7
PERIRECTAL TUBULES	12	1.8		
RECTUM	14	2.1		
RECTAL COMPLEX	11	1.7	11	1.2
FAT BODY	6.7	1.0	15	1.7
MALE GONADS	9.5	1.5		
FEMALE GONADS	39	5.9		
CARCASS	17	2.6	21	2.3

6. Different expression of 'equivalent' gene models in the two gene sets

A. TC008334 (OGS3)

TC ID	Symbol	Product	NCBI Equivalent	Fly Homologues
TC008334	LOC663766	cys-loop ligand-gated ion channel subunit	663766	Fly homologues

Beetle Paralogues
Beetle Paralogues

Adult & Larval Gene FPKMs and Enrichments ☐ SDs ☐ Whole Body

	ADULT		LARVAL	
TISSUE	FPKM	ENRICHMENT	FPKM	ENRICHMENT
HEAD	1.0	0.1	0.1	n.a.
BRAIN	2.4	0.2	1.4	n.a.
ANTERIOR MIDGUT	0.0	0.0	0.0	n.a.
POSTERIOR MIDGUT	0.0	0.0	0.1	n.a.
HINDGUT	0.9	0.1	0.0	n.a.
TUBULES (FREE)	0.0	0.0	0.0	n.a.
PERIRECTAL TUBULES	0.1	0.0		
RECTUM	0.0	0.0		
RECTAL COMPLEX	0.1	0.0	0.0	n.a.
FAT BODY	96	6.9	0.2	n.a.
MALE GONADS	221	16		
FEMALE GONADS	0.2	0.0		
CARCASS	2.9	0.2	0.0	n.a.

B. 66376 (NCBI)

NCBI ID	Symbol	Product	TC Equivalent	Fly Homologues
663766	LOC663766	cys-loop ligand-gated ion channel subunit	TC008334	Fly homologues

Beetle Paralogues
Beetle Paralogues

Adult & Larval Gene FPKMs and Enrichments ☐ SDs ☐ Whole Body

	ADULT		LARVAL	
TISSUE	FPKM	ENRICHMENT	FPKM	ENRICHMENT
HEAD	0.4	n.a.	0.1	n.a.
BRAIN	0.4	n.a.	0.3	n.a.
ANTERIOR MIDGUT	0.0	n.a.	0.0	n.a.
POSTERIOR MIDGUT	0.0	n.a.	0.0	n.a.
HINDGUT	0.1	n.a.	0.0	n.a.
TUBULES (FREE)	0.0	n.a.	0.0	n.a.
PERIRECTAL TUBULES	0.0	n.a.		
RECTUM	0.0	n.a.		
RECTAL COMPLEX	0.0	n.a.	0.0	n.a.
FAT BODY	0.1	n.a.	0.1	n.a.
MALE GONADS	0.1	n.a.		
FEMALE GONADS	0.0	n.a.		
CARCASS	0.1	n.a.	0.0	n.a.

7. Dubious reporting of tissue expression because of suspect gene models

A. TC010660

TC ID	NCBI Equivalent	Fly Homologues	Beetle Paralogues
TC010660	not available	Fly homologues	none identified

Adult & Larval Gene FPKMs and Enrichments ☐ SDs ☐ Whole Body

	ADULT		LARVAL	
TISSUE	FPKM	ENRICHMENT	FPKM	ENRICHMENT
HEAD	3.4	0.1	4.5	1.6
BRAIN	3.1	0.1	2.9	1.1
ANTERIOR MIDGUT	2.1	0.1	2.2	0.8
POSTERIOR MIDGUT	2.3	0.1	2.3	0.8
HINDGUT	2.4	0.1	1.9	0.7
TUBULES (FREE)	2.7	0.1	2.2	0.8
PERIRECTAL TUBULES	3.0	0.1		
RECTUM	2.9	0.1		
RECTAL COMPLEX	3.4	0.1	2.7	1.0
FAT BODY	34	1.1	2.1	0.8
MALE GONADS	86	2.9		
FEMALE GONADS	6.4	0.2		
CARCASS	3.3	0.1	1.8	0.7

B. 655314

NCBI ID	Symbol	Product	TC Equivalent	Fly Homologues	Beetle Paralogues
655314	LOC655314	uncharacterized protein	not available	Fly homologues	none identified

Adult & Larval Gene FPKMs and Enrichments ☐ SDs ☐ Whole Body

	ADULT		LARVAL	
TISSUE	FPKM	ENRICHMENT	FPKM	ENRICHMENT
HEAD	3.2	0.1	4.2	1.7
BRAIN	2.9	0.1	2.8	1.1
ANTERIOR MIDGUT	1.9	0.0	1.9	0.8
POSTERIOR MIDGUT	2.0	0.0	2.1	0.9
HINDGUT	2.8	0.1	1.9	0.8
TUBULES (FREE)	2.4	0.0	2.1	0.9
PERIRECTAL TUBULES	2.8	0.1		
RECTUM	2.8	0.1		
RECTAL COMPLEX	3.2	0.1	2.5	1.0
FAT BODY	98	1.9	1.9	0.8
MALE GONADS	308	6.0		
FEMALE GONADS	6.3	0.1		
CARCASS	4.7	0.1	1.7	0.7

C. TC010659

TC ID	NCBI Equivalent	Fly Homologues	Beetle Paralogues
TC010659	not available	Fly homologues	Beetle Paralogues

Adult & Larval Gene FPKMs and Enrichments ☐ SDs ☐ Whole Body

	ADULT		LARVAL	
TISSUE	FPKM	ENRICHMENT	FPKM	ENRICHMENT
HEAD	0.0	0.0	0.0	n.a.
BRAIN	0.0	0.0	0.0	n.a.
ANTERIOR MIDGUT	0.0	0.0	0.0	n.a.
POSTERIOR MIDGUT	0.0	0.0	0.0	n.a.
HINDGUT	1.1	0.0	0.0	n.a.
TUBULES (FREE)	0.1	0.0	0.0	n.a.
PERIRECTAL TUBULES	0.1	0.0		
RECTUM	0.0	0.0		
RECTAL COMPLEX	0.0	0.0	0.0	n.a.
FAT BODY	284	3.5	0.0	n.a.
MALE GONADS	865	11		
FEMALE GONADS	0.2	0.0		
CARCASS	4.4	0.1	0.0	n.a.

Expression reported by BeetleAtlas 2 for male gonads and fat body is suspect in A and B because gene models overlap that of C (*see* Figure 5D and text for details).

8. Putative retrotransposon, *Pucelle*

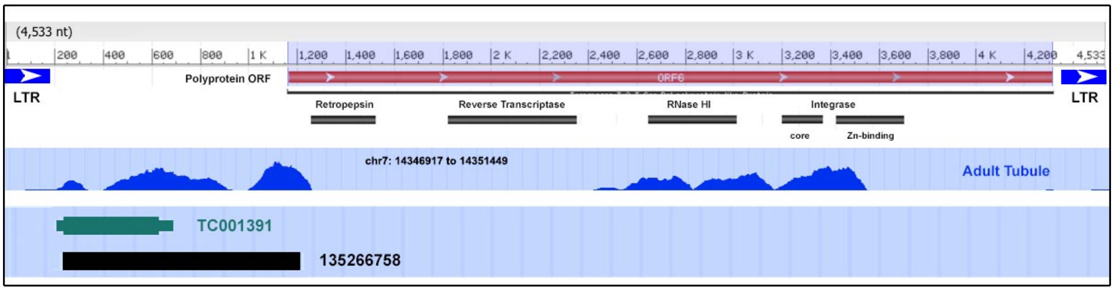

Tracks, from top to bottom: Scale (nt); Positions of LTRs and predicted polyprotein with conserved regions of genes indicated; RNA-seq reads from 14346917 to 14351449 on Chromosome 7 for adult tubule; OGS3 and NCBI gene models. We have identified fifteen instances of this transposon family, including five truncated examples and one solo LTR.

N.B. An amino sequence 99% identical to the predicted polyprotein has been deposited in GenBank as EFA13472 with the description "Transposon Ty3-I Gag-Pol polyprotein-like Protein". The associated documentation relates it to the OGS3 gene designated TC016332 which is now known to map to chromosome 4.
